# Psychometric assessment of the Persian version of the protective factors of resilience scale (PFRS)

**DOI:** 10.1002/brb3.3061

**Published:** 2023-05-28

**Authors:** Zahra Gheisari, Abbas Abdollahi, Zahra Hashemi

**Affiliations:** ^1^ Department of Educational Psychology, Faculty of Education and Psychology Alzahra University Tehran Iran; ^2^ Department of Counseling, Faculty of Education and Psychology Alzahra University Tehran Iran

**Keywords:** Iranians, protective factors, protective factors of resilience, psychometric properties, resilience

## Abstract

**Background:**

Resilience is defined as an individual's ability to recover from difficulties and overcome challenges and adversity. Recognizing and measuring internal and external protective factors have been identified as important processes for building resilience, yet to date, no valid, and reliable scales of resilience in the Persian language have been developed that recognize both internal and external protective factors.

**Methods:**

The present study was to translate the protective factors of resilience scale (PFRS) from English to Persian and analyze its psychometric properties among Iranians. Convenience sampling was used to gather data from January 2021 to February 2021 through digital internet scales, and 6 scales, including PFRS, Ryff's psychological well‐being scale, Rosenberg self‐esteem scale, life orientation test‐revised, positive and negative affect schedule, and short version of resilience scale (RS), were completed by 265 participants aged from 15 to 56. So, the aim of this study is to investigate the psychometric properties of protective factors of resilience scale among Iranians.

**Results:**

The results of the face, content, and construct validity revealed that the Persian version of the PFRS measure had acceptable validity and reliability. The Cronbach alpha for the whole scale was 0.88, and the value of the content validity index was above 0.7. A confirmatory factor analysis confirmed the three‐factor structure model of the scale (fit indices: CMIN/df = 2.51, *p* < .01; comparative fit index = 0.94, goodness of fit index = 0.90, root mean squared error of approximation = 0.07).

**Conclusion:**

In conclusion, the Persian version of the protective factors of resilience is a reliable and valid measurement to assess the protective factors and internal and external protective factors of resilience in Iranians.

## Graphical abstract

The Persian version of the PFRS is a reliable and valid measurement to assess the protective factors and internal and external protective factors of resilience in Iranians.

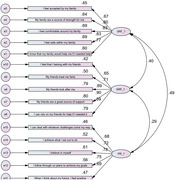

## 1 INTRODUCTION

For decades, researchers have investigated why and how can some people maintain their effectiveness in dealing with adversity and stressful situations. What factors enable them to achieve positive adaptation in this situation? Why and how some individuals are more resilient to adversity than others? (Almazan et al., 2018, 2019; Frydenber et al., 2012; Luttar, 1999; Masten, 2004; Masten et al., 1990; Mohammadinia et al., 2019; Rutter, 1990). In the field of psychology of resilience, adversity refers to the form of potentially traumatic events, difficult conditions, misfortune, stressors, and other challenging experiences (Rutter, 1990(; but due to the breadth of the concept of resilience and its application in different fields, resilience has been defined in different ways, for example, resilience has been defined as an individual's ability to adapt positively, despite threatening circumstances, such as those from adverse experiences (Howard & Johnson, 2000; Masten et al., 1990). Garmzi (1985) believed that resilience cannot be considered invulnerability to stress and adverse life conditions, but resilience is the ability to cope and recover from adverse life conditions. Fonagy et al. (1994) also described resilience as “normal growth under difficult and unfortunate conditions” (p. 233). Masten (1994) and Masten et al. (1990) also considered the distinction between the consequences of resilience and defined it in three ways: (1) showing better outcomes than expected in people at risk, (2) positive adaptation despite experiences being stressful, and (3) recovery after adverse events. American Psychology Association (2002) has defined psychological resilience as “a process of good adaptation in the face of adversity, trauma, tragedy, threats, or other significant sources of stressors such as family and relationship problems or financial problems.” Despite the multiple definitions of resilience, it can be said that the common denominator of all these descriptions is the ability to return to the initial state and adapt successfully despite high stress and adverse circumstances (Stewart et al., 1997; Brucely et al., 1998; cited in Place et al., 2002), and the distinction between different definitions is that resilience is considered capacity, process, or outcome (Masten et al., 1990). Researchers who consider resilience as an outcome emphasize the maintenance of resilience functions and skill behavior patterns or effective functions in different groups and in dealing with adverse conditions. Such consequences can be classified as good mental health, functional capacity or social skill, emotional well‐being, and so on (Olson et al., 2003). Rutter (1985, 1999) conceptualized resilience as the dynamic process involving an interaction between risk factors and both internal and external protective factors to improve the psychological outcome of a challenging condition. In other words, resilience is the use of mental processes for protecting oneself from the negative effects of stressors (Robertson et al., 2015). Therefore, it can be understood, when resilience is considered a capacity or psychological consequence, coping strategies can be considered a strategy to achieve resilience. This strategy is known as protective factors. However, in the meantime, the distinction between the process and consequences of incompatibility cannot be without ambiguity and complexity (Olsson et al., 2003).

So, in the field of resilience, it is important to recognize the protective factors that encourage resilience. Rutter (1990) defined protective factors as features and variables that balance, improve, or change persons’ responses in high‐risk environments that are likely for adverse consequences. According to Garmezy (1985), protective factors fall into three categories: (1) individual factors, (2) family factors, and (3) community‐based factors. Individual protective factors can include biological, psychological, and personality traits. Past and contemporary studies of resilience have revealed that the number of individual factors one has is associated with how resilient he/she will be (Garmezy, 1985; Luthar, 1991, 1999; Rutter, 2012; Yates & Masten, 2004). Family protective factors are those that increase one's ability to deal with undesirable conditions through the use of family factors. Such factors include parenting style, relationships among family members, socioeconomic status, and level of attachment (Gizir, 2004; Yates & Masten, 2004). It may be that families have a protective mechanism as a source of social support (Elashi et al., 2020; Nasrabadi et al., 2021). Protective factors at the community level include factors such as social institutions, religious centers, peer groups, and positive relationships with neighbors (Brooks, 2006; Gizir, 2004; Taylor, 2007; Vincent, 2007; Yates & Masten, 2004). These three levels have an important influence on the individual as recognized in the socio‐ecological literature (Allen & Kern, 2017, 2019; Allen et al., 2016, 2018; Bronbrenner, 1979). Having any of these protective factors (e.g., a supportive family, greater socioeconomic status, and positive peer groups) can help people adapt to stressful events and also recover from traumatic circumstances in their lives (Dias & Cadime, 2017; Hamby et al., 2018). Additionally, these factors contribute to an individual's healthy psychological functioning and well‐being (Hjemdal et al., 2006).

This view of resilience is related to the variable‐oriented approach, in which the investigation of factors and factors that protect resilience is proposed. According to Masten and Reed (2002), two main approaches can be seen in the field of resilience research: (1) variable‐based approach and (2) person‐based approach. The variable‐based approach examines the relationship among individual characteristics, environment, and dangerous experiences. In fact, this model seeks to find certain protective factors for different aspects of adaptation and effective adaptation. The person‐based approach involves the discrimination and comparison of resilient and vulnerable groups who demonstrate adaptive and maladaptive outcomes within the same high‐risk circumstances (Masten & Reed, 2002).

In line with the variable‐based approach, researchers have focused on measuring variables and factors that affect resilience. Through this approach, for identify individual, familiar, and social protective factors, and to examine the relationship between them, several questionnaires and scales have been developed, including the Connor‐Davidson resilience scale (Connor & Davidson, 2003), the resilience scale (RS; Wagnild & Young, 1993), and the brief resilience scale (Smith et al., 2008).

Three review studies have been conducted to study the measures and scales developed to assess resilience (Harms et al., 2017; Pangallo et al., 2015; Windle et al., 2011), in which all concluded most resilience measures have concentrated on personal factors (e.g., personal traits and characteristics). Harms et al. (2017) argued that there are two major problems with resilience measures that focus heavily on personal factors. First, measures can be lengthy for what they intend to measure and second, measures that sum items into a single score do not recognize the true multidimensionality of protective factors related to resilience.

Due to these deficiencies, the protective factors of resilience scale (PFRS) was developed by Harms et al. (2017) to assess not only personal protective factors but also the role of peers and family as protective factors. The model consisted of three subscales with five items each: personal resources (PR), family resources (SR‐F), and peer resources (SR‐P). This scale was also validated in Spain (León et al., 2020) and found to be valid and reliable in both the Spanish general and chronically ill populations.

Despite resilience's importance to the individual, the most used measures in Iran are CD‐RISC ((Connor & Davidson, 2003) and the short form of RS (RS‐14; Wagnild, 2009)), which have some deficiencies, as mentioned earlier. Thus, there are few scales in Iran to measure both internal and external protective factors of resilience. In another word, none of the currently used scales in Iran measure and examine protective factors and only deal with the overall level of resilience in the individual.

Therefore, the main hypothesis of this research is that “the PFRS is valid and reliable in the Persian language and among the Iranian population.”

## 2 METHOD

### 2.1 Participants

Participants consisted of 265 Iranians who were invited to participate in the study and complete the scales through social networks like Telegram and WhatsApp applications. The only inclusion criterion was to be Iranian and all of the 265 participants who completed the forms were Iranian. This study had one exclusion criterion too, which was being under the age of 15. All the participants were 15 or above as well. So, none of them was removed due to exclusion criteria.

### 2.2 Procedure

The (Brislin, 1986) method of translation was employed to translate the PFRS measure into Persian. Two translators separately translated the PFRS to Persian and were fluent in Persian and English. The first translator was asked to translate the measure from English to Persian. Then, the second translator was asked to back‐translate the Persian version to English without seeing the original English items. The back‐translated English version was sent to the PFRS developers (Julie Ann Pooley and Craig Harms), who confirmed that there were no significant differences between the original PFRS and the back‐translated version.

Convenience sampling was used to gather data from January 2021 to February 2021 through digital internet questionnaires. The scales were sent by various messaging apps, including WhatsApp and Telegram, which asked people to complete the survey and send the survey link to their relatives and friends to be completed. Through this process, a total of 265 individuals filled out the scales.

### 2.3 Instruments

#### 2.3.1 Protective factors of resilience scale (PFRS)

This scale was developed by Harms et al. (2017) to assess the protective factors of resilience. A sample of university students was used to study the factor structure and a second sample of the community population to confirm it. Finally, there was a second‐factor model, which consisted of three subscales, each one including 5 items (15 items total): PR, family resources (SR‐F), and peer resources (SR‐P). The scale has a seven‐point Likert response scale ranging from one (totally disagree) to seven (totally agree). High scores indicate high resources on each subscale. A high Cronbach alpha coefficient (.93) was shown in PFRS. Moreover, by investigating the relations among different variables (coping styles, self‐esteem, and life satisfaction), good evidence of construct validity emerged. As mentioned before, this was formerly validated in Spain (León et al., 2020) and found to be valid and reliable in both the Spanish general and chronically ill population. In this study, five measures were used along with the PFRS measure.

#### 2.3.2 Ryff's psychological well‐being (PWB) scale

The original version of this scale consists of 120 items and has an internal consistency of 0.84 (Ryff, 1989). For the present study, a short form consisting of 18 items rated on a 6‐point Likert scale ranging from 1 (totally disagree) to 6 (totally agree) was used. This measure has six subscales, including self‐acceptance, positive relationships, autonomy, environmental mastery, purpose in life, and personal growth. The Persian version of this measure has been validated (Khanjani et al., 2014), with Cronbach's alpha coefficients of the subscales ranging from .52 to .76 and .71 for the total scale.

#### 2.3.3 The Rosenberg self‐esteem scale (RSES)

This scale was developed by Rosenberg (1965), and the Persian version was validated by Rajabi and Bohlool (2007). This instrument contains 10 items rated on a 4‐point Likert scale ranging from 1 (strongly disagree) to 4 (strongly agree), and 5 items are reversed coded. Total scores range between 10 and 40, with higher scores indicating higher self‐esteem. The Cronbach alpha coefficient of the Persian version was .84 (Rajabi & Bohlool, 2007).

#### 2.3.4 The short version of the resilience scale (RS‐14)

This instrument was initially developed by Wagnild and Young (1993) and consists of 25 items; later the short version of this measure was introduced (Wagnild, 2009). The short version includes 14 items rated on a 5‐point Likert scale ranging from 1 (totally disagree) to 5 (totally agree). The Persian version was validated in Iran and had an internal consistency of 0.78 (Hashemi et al., 2018).

#### 2.3.5 The life orientation test‐revised (LOT‐R)

This scale consists of 10 items rated on a 5‐point Likert scale ranging from 1 (totally disagree) to 5 (totally agree; Scheier et al., 1994). Three items evaluate optimism and three items evaluate pessimism. The remaining four items are considered “fillers.” The internal consistency coefficients of optimism and pessimism in the Persian version were .88 and .77, respectively (Khodaei et al., 2016).

#### 2.3.6 The positive and negative affect schedule (PANAS)

This scale was developed by Watson et al. (1988), and the Persian version was validated in Iran (Mohammadi, 2011). There are 20 items in this measure rated on a 5‐point Likert scale ranging from 1 (very slightly or not at all) to 5 (extremely), which indicate the level of each positive or negative affect experienced. Ten items evaluate positive affect and the other 10 items evaluate negative affect. The Cronbach alpha coefficients for this measure were between .82 and .88 in the Persian version.

### 2.4 Face validity

To assess the face validity of the Persian version of the PFRS, 8 individuals from different ages (ranging from 19 to 56) and different academic education levels (bachelor, master, and doctoral degrees) were asked to evaluate each of the items regarding their difficulty, ambiguity, and relevancy on a 5‐point Likert scale from 1 (completely difficult/ambiguous/irrelevant) to 5 (completely simple/clear/relevant). To evaluate the quantitative face validity, the following formula was employed:

$$\mathrm{Quantitative}\;\mathrm{face}\;\mathrm{validity}\;=\;\mathrm{importance}\;\times \;\mathrm{frequency}\left(\%\right)$$

In this formula, frequency refers to the number of participants who reported a score of 4 or 5 for the indicator, and importance refers to the mean score of the indicator. According to Hajizadeh and Asghari (2011), if the score for each indicator is equal to or greater than 1.5, that indicator is considered acceptable and remains on the scale.

### 2.5 Content validity

The qualitative content validity was assessed by 10 expert psychologists. They were requested to comment on the grammar and proper placement of phrases. Some items changed due to the experts’ comments. The quantitative content validity was assessed by the same experts using the content validity ratio (CVR) and content validity index (CVI) (Cook & Beckman, 2006). The experts were asked to score the essentiality of the items based on a 3‐point Likert scale (1: not essential, 2: useful but not essential, and 3: essential). The following formula was used to assess the CVR for each of the items:

$$\mathrm{CVR}=\frac{ne-N/2}{N/2}$$

In this formula, *N* is the number of the experts, and *ne* is the number of those experts who reported a score of 3 for the item.

The CVI was used to evaluate the simplicity, relevancy, and clarity of each item from the experts’ perspective. Each item was assessed on a 4‐point Likert scale (e.g., 1: not relevant at all, 2: somewhat relevant, 3: quite relevant, and 4: highly relevant). The following formula was used to assess CVI:

$$\mathrm{CVI}=\frac{n\left(3\;or\;4\right)}{N}$$

CVI is calculated by taking the total number of experts who scored 3 or 4 on the item divided by the total number of experts (in this case, 10) (Lynn et al., 2006). If the CVI value is 0.7 or more, the item is acceptable and remains in the measure (Polit et al., 2007).

### 2.6 Construct validity

For assessing construct validity, a preliminary analysis was conducted, the results of which are reported in Section 3. To assess the structural validity of the three factors of the PFRS (each including five items), a confirmatory factor analysis (CFA) with maximum likelihood estimation was conducted in AMOS 24 software.

According to Plichta and Kelvin (2015), the acceptable sample size for conducting CFA is equal to 5–20 times more than the measure's items. As the PFRS contains 15 items, the sample size should be between 150 and 300. The sample size of 265 satisfied this requirement. First, factor loadings of the indicators and, then, the measurement fitting indices were checked.

To assess the convergent validity of the three factors of the PFRS measure, the average variance extracted (AVE) was used. If the AVE value is greater than 0.5, the variable has acceptable convergent validity. The AVE calculated as follows: total of the squared multiple correlations plus the total sum of each variable then divides it by the number of factors in that variable. The Cronbach alpha coefficient and the composite reliability (CR) value were used to assess the reliability and the internal consistency of the measure. CR calculated with this formula (Tabachnick & Fidell, 2013):

$$\mathrm{CR}\;=\;\frac{\left(\sum \mathrm{factor}\;\mathrm{loading}\right){\;}^{2}\;}{\left(\sum \mathrm{factor}\;\mathrm{loading}\right){\;}^{2}\;+\;\left(\sum \mathrm{seasur}\;\mathrm{sent}\;\mathrm{error}\right)}$$

According to Tabachnick and Fidell (2013), if the CR and Cronbach's alpha values are greater than .7, a variable has acceptable CR.

### 2.7 Convergent and divergent validity

According to previous studies (Harms et al., 2017; León et al., 2020), Ryff's psychological well‐being (PWB) scale, the short version of the RS‐14, the Rosenberg self‐esteem scale (RSES), the subscale of the positive affects positive and negative affect schedule (PANAS) measure, and the optimism subscale of the Life Orientation Test (LOT) were used to assess the convergent validity of the PFRS. Moreover, the negative effects subscale of the PANAS measure and the pessimism subscale of the LOT measure were used to assess the divergent validity of the PFRS (Harms et al., 2017; León et al., 2020).

## 3 RESULTS

A total of 265 participants with a mean age of 31.7 (SD = 9.32, range: 15–56) filled the scales. They had different academic degrees: 20% (*n* = 53) reported having a high‐school diploma or less, 41.1% (*n* = 109) reported a bachelor's degree, 31.7% (*n* = 84) reported having a master's degree, and 7.2% (*n* = 19) a doctoral degree.

### 3.1 Face and content validity

As Table 1 shows, the face validity scores for all items were greater than 1.5 (ranging from 2.18 to 5). As such, all indicators were kept in the PFRS measure.

**TABLE 1:** Face validity, content validity index (CVI), means, and standard deviations of the protective factors of resilience scale (PFRS) items.

No	Factor	Items	Face validity			CVI			CVR	Mean	Std. deviation
			Simplicity	Relevancy	Clarity	Simplicity	Relevancy	Clarity	Essential		
1	PR	I can deal with whatever challenges come my way	4.04	3.93	3.93	1	0.8	1	1	5.27	1.34
2	PR	I achieve what I set out to do	4.75	3.93	4.87	1	0.8	1	0.8	5.01	1.36
3	SR‐P	I feel that I belong with my friends	5	2.18	3.82	1	0.8	1	0.8	5.45	1.48
4	SR‐F	My family is a source of strength for me	3.71	4.62	3	1	1	1	0.8	5.88	1.29
5	PR	I believe in myself	3.93	4.04	4.62	1	1	0.8	1	5.62	1.39
6	PR	I follow through on plans to achieve my goals	3.93	4.87	4.62	1	1	1	0.8	5.33	1.40
7	SR‐P	My friends treat me fairly	2.81	2.81	2.9	1	0.8	1	0.6	5.94	1.19
8	SR‐F	I feel accepted by my family	4.87	4.75	4.87	1	1	1	1	5.80	1.34
9	SR‐P	My friends look after me	4.87	3.93	4.75	1	1	1	0.8	5.28	1.48
10	SR‐F	I know that my family would help me if I needed help	5	5	5	1	1	1	0.8	6.29	1.08
11	SR‐P	My friends are a great source of support	3.93	4.8	4.87	1	1	1	0.8	5.04	1.57
12	SR‐F	I feel comfortable around my family	4.87	3.82	5	1	1	1	0.8	5.92	1.36
13	PR	When I think about my future, I feel positive	4.75	4.75	3.93	1	1	1	0.8	5.86	1.39
14	SR‐P	I can rely on my friends for help if I needed it	4.62	4.8	5	1	1	1	0.8	5.26	1.51
15	SR‐F	I feel safe within my family	4.87	5	5	1	1	1	1	6.25	1.13

Abbreviation: PR, personal resources.

Moreover, the values of the CVR for all the items were greater than 0.6 (the acceptable value with 10 experts in the Lawshe table), which indicated that all of the items had acceptable content validity (Lawshe, 1975).

The CVI values for all of the items were greater than 0.7 and were therefore included in the survey, as well. The mean and standard deviations for each indicator are shown in Table 1 as well.

### 3.2 Construct validity

#### 3.2.1 Preliminary analysis

A missing value analysis showed no missing data in the dataset. The results of normality analysis in the AMOS software (version 24) showed that the kurtosis values ranged from −0.12 to 4.80 and skewness values ranged from −1.94 to −0.5, which indicated that the data was normally distributed. Kurtosis and skewness values, being within ±5 and ±2, respectively, demonstrate a normally distributed dataset (Tabachnick & Fidell, 2013).

#### 3.2.2 Confirmatory factor analysis

**FIGURE 1:**
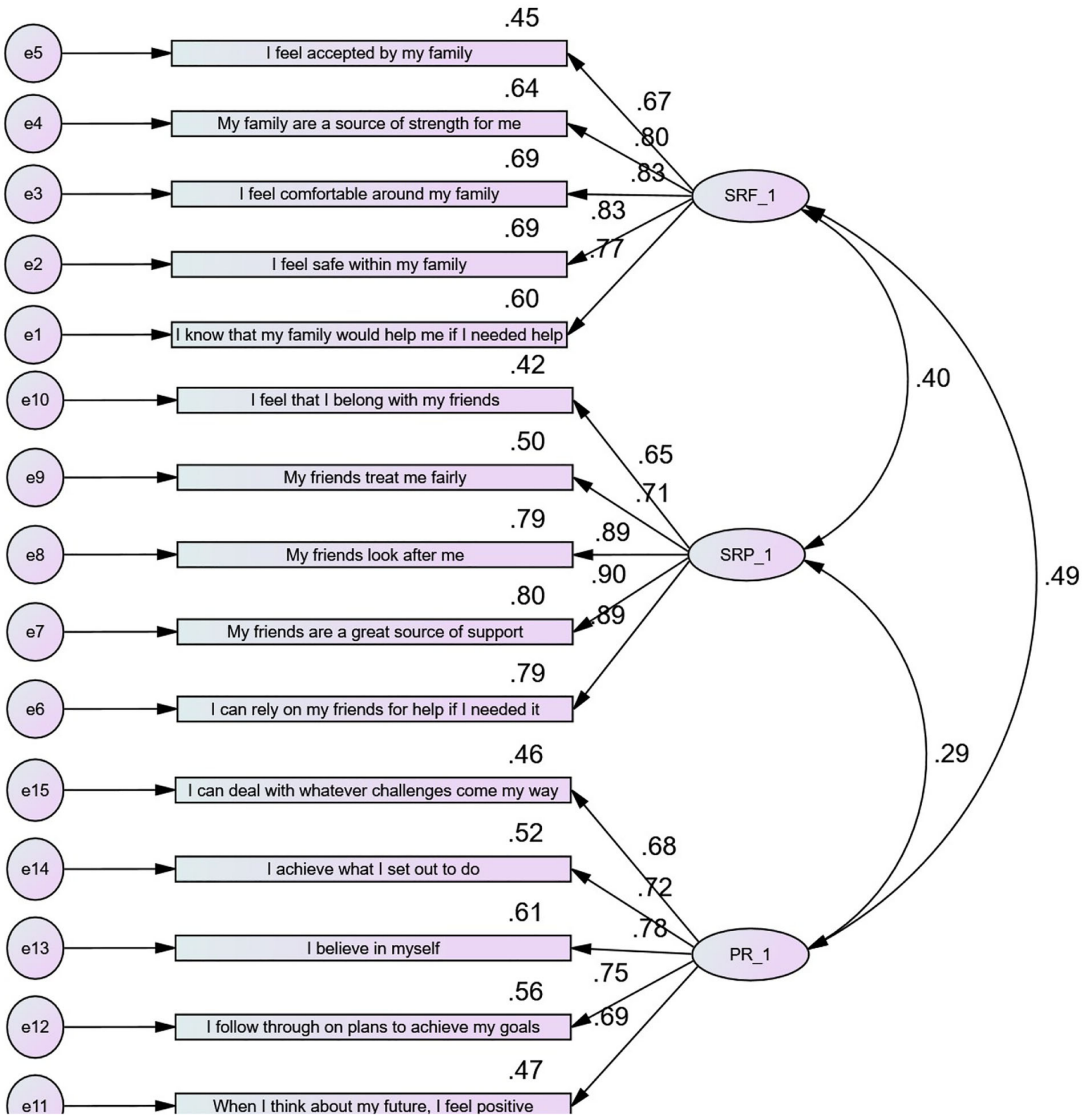
Confirmatory factor analysis with factor loadings for the three subscales of protective factors of resilience scale (*p* < .001).

First, factor loadings of the indicators were checked. According to Kline (2015), if the factor loading of an indicator is greater than 1, negative, or less than 0.4, this indicator must be removed. No factor violated this criterion, and the relationships among all indicators with the latent variables were significant (*p* < .001). Thus, all indicators remained in the PFRS measure (see Figure 1). Moreover, the results of CFA confirmed that the PFRS serves as an effective indication of three factors of protective factors for resilience (PR, family resources, and peers resources). Correlation analyses showed that the relationships among all three subscales were positive and significant (*p* < .001) (see Figure 1). The highest correlation coefficient was between family and PR (*r* = .49), and the lowest correlation coefficient was between peers and PR (*r* = .29) (see Figure 1 in appendix A).

Next, the measurement fitting indices were checked. Fitting indices included CMIN/df < 5; comparative fit index (CFI) >0.90; goodness of fit index (GFI) >0.90; normed fit index (NFI) >0.90; Tucker–Lewis index (TLI) >0.90; and root mean squared error of approximation (RMSEA) <0.08 (Byrne, 2012). The results of the measurement fitting indices showed that the three factors of the PFRS measure adequately fit the data (CMIN/df = 2.51, *p* < .01; CFI = 0.94, GFI = 0.90, NFI = 0.90, TLI = 0.93, RMSEA = 0.07).

In the third step, Cronbach's alpha coefficients, convergent validity, and CR were calculated. As Table 2 shows, the results indicated that all three factors of the PFRS measure possessed adequate construct reliability and validity.

**TABLE 2:** Average variance extracted (AVE), composite reliability (CR), and Cronbach's alpha coefficient for the protective factors of resilience scale (PFRS) and its subscales.

Variable	AVE	CR	Cronbach's alpha
Family resources	.61	.84	.88
Peers resources	.66	.83	.90
Personal resources	.52	.74	.84
PFRS	.60	.90	.88

### 3.3 Convergent and divergent validity

The correlations between the PFRS measure (and its three factors) and the other measures in the study were assessed, including the short form of the RS‐14, Ryff's PWB scale, and some of its subscales (self‐acceptance, positive relations, autonomy, environmental mastery, personal growth, and purpose in life), the subscale of the positive affects PANAS measure, the optimism subscale of the LOT, and the RSES (see Table 3) had positive relationships with the PFRS measure, which shows the convergent validity of the PFRS scale. The correlation analysis also showed significant and negative associations between the PFRS measure and the negative affects subscale of the PANAS measure and the pessimism subscale of the LOT measure that confirm the divergent validity of the scale (see Table 3).

**TABLE 3:** Pearson's correlations among protective factors of resilience scale (PFRS), the three factors, and other instruments.

Measures/subscales	SR‐F	SR‐P	PR	PFRS
PWB (psychological well‐being) total	0.35^**^	0.33^**^	0.60^**^	0.56^**^
PWB self‐acceptance	0.34^**^	0.20^**^	0.61^**^	0.50^**^
PWB positive relations	0.31^**^	0.52^**^	0.27^**^	0.50^**^
PWB autonomy	0.02	0.02	0.35^**^	0.17^**^
PWB environmental mastery	0.36^**^	0.21^**^	0.48^**^	0.45^**^
PWB personal growth	0.20^**^	0.21^**^	0.50^**^	0.40^**^
PWB purpose in life	0.01	−0.04	0.002	−0.01
Resilience Scale‐14	0.30^**^	0.26^**^	0.73^**^	0.56^**^
Life Orientation Test (LOT)‐optimism	0.35^**^	0.18^**^	0.50^**^	0.44^**^
LOT‐pessimism	−0.09	−0.19^**^	−0.18^**^	−0.21^**^
PANAS‐positive affects	0.31^**^	0.24^**^	0.55^**^	0.48^**^
PANAS‐negative affects	−0.35^**^	−0.26^**^	−0.43^**^	−0.45^**^
Self‐esteem	0.33^**^	0.20^**^	0.66^**^	0.51^**^

*Note*: ** indicates significance at the level of 0.01.

Abbreviations: PR, personal resources; SR‐F, family resources, SR‐P, peer resources.

## 4 DISCUSSION

The purpose of this study was to verify the psychometric properties of the PFRS in a sample of the Iranian population. The final results showed that the translated Persian version demonstrated satisfactory face and content validity as well as the English and Spanish versions (Harms et al., 2017; León et al., 2020). A CFA supported the three‐factor structure of the PFRS as was demonstrated in the original study conducted by Harms et al. (2017). The three factors of the scale included PR (as an internal protective factor), family resources, and peer resources (both as protective external factors).

The factor loading values were all greater than 0.5 (ranging from 0.65 to 0.90; see Figure 1 in appendix A), so all items were kept in the scale. The Cronbach alpha and CR coefficients showed that the three factors of the PFRS have an acceptable internal consistency. Moreover, the AVE coefficients showed that each factor has a suitable internal correlation (see Table 2).

PFRS's divergent and convergent validity were confirmed, as the scale factors were significantly related to other measures that assess similar constructs. For instance, the PR factor was highly positively correlated (*r* ≥ .5) with the RS‐14 measure (which only measures the personal factors related to resilience), RSES, the Ryff subscales of self‐acceptance and personal growth, optimism (LOT‐O), and PANAS. The correlation analysis also showed that there are significant negative relationships between the PFRS measure and both pessimism (LOT‐P) and negative effects from the PANAS measure. These results are consistent with the previous study on PFRS in Spain (León et al., 2020).

This study has multiple implications. First, the Persian version of the PFRS demonstrated adequate psychometric properties; thus, it is a valid and reliable scale to evaluate both internal and external protective factors in Iranian individuals. This implication is consistent with the findings of other research on this scale (Harms et al., 2017; León et al., 2020). Second, this scale is much shorter than many of the scales that have evaluated this factor (Windle et al., 2011). Many kinds of research that are based on filling out scales or questionnaires are not completed by many people due to their length. This might cause serious problems for researchers in collecting the sample or may affect contributors’ focus while answering questions; therefore, such short scales may be more attractive for researchers and contributors.

This study also had some limitations. One of these limitations is that the PFRS was taken by only individuals without any chronic disease, and the mean of the resiliency in the sample was high. This limitation was due to the condition of COVID‐19 that limited our access to this particular sample. Other studies would be necessary to investigate PFRS's validity and reliability in those populations in better conditions with respect to the current pandemic.

Another important limitation of this study is that because of the convenience sampling, the results of this research cannot be generalized to the entire society with certainty and caution should be observed.

In conclusion, the findings of this study support the three‐factor structure factors of the PFRS, and based on the findings, the PFRS seems to be a valid and reliable scale for Iranians. Moreover, due to the scales’ brevity and its capability to assess both internal and external protective factors of resilience, it may be better to other measures that are available for the Persian population. Nevertheless, it would be preferable to extend this work with a conclusive sample and to people with mental health problems or a chronic disease before it can be implemented on a wider scale.

Moreover, as PWB is positively correlated with protective factors of resilience, it seems that maybe resilience training and informing people about the importance of the protective factor would improve PWB.

Moreover, the positive correlation between optimism (subscale of LOT) and positive affects (subscale of PANAS) with PFRS and negative correlation between pessimism (subscale of LOT) and negative affects (subscale of PANAS) with this scale may mean that low levels of protective factors of resilience would cause negative emotions and pessimism. So by teaching people about the protective factors of resilience and its importance for strengthening them, they probably become more optimistic and may experience positive emotions more than before.

## CONFLICT OF INTEREST STATEMENT

The authors declare that they have no conflict of interest.

### PEER REVIEW

The peer review history for this article is available at https://publons.com/publon/10.1002/brb3.3061.

## INFORMED CONSENT

Informed consent was obtained from all individual participants included in the study.

## Data Availability

The datasets (SPSS and AMOS files) generated and/or analyzed during the current study are available from the corresponding author upon reasonable request.
